# Epigenome-wide analysis of sperm cells identifies *IL22* as a possible germ line risk locus for psoriatic arthritis

**DOI:** 10.1371/journal.pone.0212043

**Published:** 2019-02-19

**Authors:** Remy A. Pollock, Laila Zaman, Vinod Chandran, Dafna D. Gladman

**Affiliations:** 1 Psoriatic Arthritis Program, Centre for Prognosis Studies in the Rheumatic Diseases, Krembil Research Institute, University Health Network, Toronto, ON, Canada; 2 Division of Rheumatology, Department of Medicine, Faculty of Medicine, University of Toronto, Toronto, ON, Canada; 3 Institue of Medical Science, University of Toronto, Toronto, ON, Canada; 4 Department of Laboratory Medicine and Pathobiology, Faculty of Medicine, University of Toronto, Toronto, ON, Canada; University of Bonn, Institute of Experimental Hematology and Transfusion Medicine, GERMANY

## Abstract

Psoriasis and its associated inflammatory arthritis, psoriatic arthritis (PsA), have a clear heritable component, but a large proportion of the heritable risk remains unexplained by gene sequence variation. This study aimed to determine if epigenetic factors contribute to the missing heritability in psoriatic disease. DNA methylation profiling was performed on sperm cells from 23 probands with psoriasis without PsA (PsC), 13 PsA probands, and 18 unaffected controls. Differentially methylated CpGs and regions (DMRs) were identified and validated by pyrosequencing. Underlying AluY and copy number variation (CNV) in the *HCG26* and *IL22* genes, respectively, were assessed by genotyping. Array, subject’s age, age of psoriasis onset, psoriasis severity, and medication usage were found to influence methylation at many genes and were included as covariates in the analysis. Between PsC probands vs. controls, 169 DMRs were found; 754 DMRs were found between PsA probands vs. controls, and 86 between PsA and PsC probands (adjusted p<0.05). Differences in methylation across DMRs were generally subtle (<10%) but correlated well with pyrosequencing. Biological inference prioritized notable DMRs associated with skin disease (*SIGLEC14*, *JAM3*, *PCOLCE*, *RXRB*), skin and/or joint disease (*MBP*, *OSBPL5*, *SNORD115*, *HCG26*), and joint disease (*IL22*, *ELF5*, *PPP2R2D*, *PTPRN2*, *HCG26*). Hypermethylation of the DMR within the first exon of arthritis-associated *IL22* showed significant correlation (rho = 0.34, 95% CI 0.06–0.57, p = 0.01) between paired sperm and blood samples, independent of a CNV within the same region. Further studies are needed to rule out underlying genetic causes and determine if these represent heritable, constitutional epimutations, or are the result of exposure of germ cells to endogenous or exogenous environmental factors.

## Background

Psoriasis is a common inflammatory skin disease associated with significant morbidity, mortality, and poor quality of life that affects approximately 3% of Caucasians [1, 2]. Approximately 30% of psoriasis patients develop psoriatic arthritis (PsA), an inflammatory arthritis characterized by peripheral and/or axial arthritis, skin and nail disease, dactylitis, and enthesitis. The high recurrence risk ratios among first-degree relatives of psoriasis and PsA patients [3, 4], and higher disease concordance among monozygotic (62–70%) than dizygotic twins (21–23%) [5–8] suggest that both have a strong heritable component. Numerous susceptibility loci for psoriasis and PsA have been identified [9]; however together they account for only ~40% of the heritability of psoriasis and PsA [10].

The paradigm of DNA sequence variation as the sole substrate of heritability is challenged by animal studies demonstrating the ability of epigenetic marks to be inherited [11], as in the examples of agouti and axin mice, which carry DNA methylation variations within retrotransposons inserted into the agouti viable yellow (*A*^*vy*^) and axin fused (*Axin*^*FU*^) alleles, respectively, and result in variable expressivity of agouti and axin loci leading to variable coat colour [12] and kinked-tail [13] phenotypes. Moreover, methylation status of the *Axin*^*FU*^ allele can be inherited through both maternal and paternal transmissions; however, the penetrance of the kinked-tail phenotype is higher following paternal transmission [13]. The methylation status of the *Axin*^*FU*^ allele in somatic tissues is also reflected in sperm cells. These studies suggest that epigenetic marks can be inherited due to a failure to reset methylation of *A*^*vy*^ and *Axin*^*FU*^ in the germ line, and this resistance to resetting can result in a parental transmission bias or ‘parent-of-origin’ effect.

Parent-of-origin effects (POE) have been identified in humans in epidemiological analyses of multiple complex diseases including psoriasis and PsA. Several independent investigations, including studies of large psoriasis and PsA cohorts from the Faroe Islands, Scotland, and Canada [14–17], have consistently demonstrated a greater prevalence of psoriasis among the offspring of psoriatic fathers compared to psoriatic mothers, and a significantly greater tendency for psoriasis and PsA probands to report an affected father compared to an affected mother. Paternal transmission is accompanied by a significant reduction in age of psoriasis onset, and a tendency to manifest as the more severe PsA phenotype in subsequent generations.

Evidence of POE in psoriasis and PsA suggests that epigenetic mechanisms may contribute to the missing heritability in psoriatic disease. POE in humans may be mediated by epigenetic defects with or without an underlying genetic cause (known as secondary and primary epimutations, respectively [18, 19]). The aim of this study was to test the hypothesis that epigenetic marks contribute to the heritability of psoriatic disease by performing an epigenome-wide analysis of sperm cells from probands with PsA, cutaneous psoriasis without PsA (PsC) and controls to identify putative heritable DNA methylation variants. The second aim was to determine if these marks are present in somatic cells (whole blood) from the same patients and controls, and investigate the impact of underlying genetic variation on aberrant methylation marks.

## Materials and methods

### Samples

Male PsC and PsA probands were recruited from the University of Toronto Psoriatic Disease Program. All PsC patients were diagnosed by a dermatologist and examined by a rheumatologist to verify the absence of PsA. All PsA patients were diagnosed by a rheumatologist and satisfied the CASPAR criteria [20]. Unaffected male controls with no family history of PsC or PsA were recruited from the general population. All participants provided written informed consent and the study was conducted with approval from the University Health Network Research Ethics Board. Participants provided semen samples from which the motile fraction of mature spermatozoa was isolated by two-layer density gradient centrifugation using ISolate reagent (Irvine Scientific, Santa Ana, CA, USA) according to the manufacturer’s instructions.

### DNA extraction and bisulfite conversion

Somatic cells were removed using a solution of 0.5% Triton X-100 and 0.1% SDS. Sperm cells were lysed with 400ul of 100mM Tris-Cl (pH 8), 10mM EDTA, 500nM NaCl, 1% SDS, and 2% B-mercaptoethanol and 100ul of Proteinase K (20mg/ml) at 55°C and 900rpm. An additional 50ul of Proteinase K was added after 2 hours, and again after 18 hours. After 20 hours of incubation, 20μl of RNase A/T1 and 10μl RNAse H were added and the samples incubated at 37°C for 30 minutes. DNA was extracted by phenol-chloroform extraction and quantity and purity were assessed by NanoDrop spectrophotometry. DNA (500ng) was treated with sodium bisulfite using the EZ DNA Methylation Kit (Zymo Research, Irvine, CA, USA) according to the manufacturer’s instructions for Illumina Infinium Methylation Assays.

### Epigenome-wide methylation analysis

Bisulfite converted DNA samples were interrogated on Infinium HumanMethylation 450k v1 BeadChips (Illumina, San Diego, CA, USA) according to the manufacturer’s protocol. Arrays were scanned on the iScan system (Illumina, San Diego, CA, USA). Fluorescence intensities were quantified, and quality control was performed in GenomeStudio Version 2011.1 (Illumina, San Diego, CA, USA) using the HumanMethylation450_15017482_v.1.2 annotation file. Data were normalized against controls and background subtracted. The datasets generated and analysed during the current study are available in the Gene Expression Omnibus (GEO) repository (accession number GSE126017).

### Bioinformatics and statistical analyses

Data were imported into the lumi package for colour balance adjustment and quantile normalization [21–23]. Type I vs II probe bias was corrected using the BMIQ method implemented through ChAMP [24]. A total of 485,577 CpG sites were initially assessed on the arrays. Probes were filtered based on detection p value (>0.01), cross-hybridization to multiple genomic locations, and presence of SNPs (MAF>5%) at the CpG or single base extension site. Furthermore, the least variable 25% of probes based on interquartile range (IQR) were removed [25, 26]. After filtering, 331,193 CpG sites were carried forward for analysis. The DMRcate package was used to identify contiguous genomic regions that differ between groups, or differentially methylated regions (DMRs), by kernel smoothing [27]. Comparisons planned *a priori* were: PsC probands vs. controls, PsA probands vs. controls, and PsA vs. PsC probands. DMRs were determined by linear regression with adjustment for clinical and technical covariates which significantly affected methylation. WebGestalt [28] was used to perform Overrepresentation Enrichment Analysis of gene ontologies, pathways, diseases, phenotypes, and chromosomal regions. Lists of DMRs from each comparison were tested for enrichment against a reference list of all of the CpGs analyzed. Enriched chromosomal regions were visualized using Integrated Genomics Viewer v2.3.

### Pyrosequencing

For technical validation of array results, *TPPP* (cg22936884), *CSMD1* (cg12027248), *PTDSS2* (cg22993527), and *HCG26* (cg16166826) methylation was assessed in bisulfite converted sperm DNA samples on a PyroMark Q24 system. For whole blood validation, whole blood DNA was extracted using a modified salting out technique (Gentra PureGene Blood kit), bisulfite converted, and methylation was measured at *PTPRN2*, *ELF5*, *IL22*, *OSBPL5*, *H19*, *MBP*, *SNORD115*, *CARS2*, and *JAM3* as described above.

### Transposable element and CNV genotyping

Genotyping of the 298bp *HCG26* AluY insertion was performed by amplifying the surrounding region using primers flanking the insertion site (S1 Table). PCR was performed using 1X PCR buffer (Quanta Biosciences Inc.), 1.0mM MgCl_2_, 200uM dNTPs, 500nM forward and reverse primers, 1U AccuStart II Taq polymerase (Quanta), and 100ng of DNA. Products were amplified on a 2720 thermal cycler (Applied Biosystems) at 94°C for 1 minute, 30 cycles of 94°C for 30 seconds, 60°C for 30 seconds, and 72°C for 90 seconds. Alleles containing the insertion yielded a 1697bp product, while alleles without the insertion yielded a 1399bp product, which were resolved on a 1.5% agarose gel. Genotyping of the *IL22* CNV was performed using an inventoried Taqman Copy Number Assay (Hs00146600_cn), Taqman RNAseP Copy Number Reference Assay, and 1X Taqman Genotyping Master Mix (Life Technologies) following the manufacturer’s instructions and cycling conditions. Assays were performed in quadruplicate on a 7900HT system and analyzed using CopyCaller v2.1 software (Life Technologies).

## Results

### Investigation of clinical and demographic factors that affect sperm methylation

Following quality control, 54 subjects (23 PsC, 13 PsA probands, and 18 unaffected controls) were included in the analysis (Table 1). Clinical, demographic, and technical characteristics affecting methylation across all CpG sites were explored by linear regression. Methylation levels at many genes were significantly associated with array chip, subject’s age, age of psoriasis onset, Psoriasis Area and Severity Index (PASI- a clinical measure of psoriasis severity), and medication usage (non-steroidal anti-inflammatory drugs, disease modifying anti-rheumatic drugs, or biologics) (S1 Fig). For this reason, in the planned comparisons of PsC patients vs. controls and PsA patients vs. controls, age and slide were included as covariates, while in the comparison of PsA vs. PsC, age, slide, medications, age of psoriasis onset, and PASI were included as covariates.

**Table 1:** Demographic and clinical characteristics of the study subjects.

Variable	PsC n = 23 # (%) or Mean (SD)	PsA n = 13 # (%) or Mean (SD)	Controls n = 18 # (%) or Mean (SD)	P Value^†^
Males	100%	100%	100%	n/a
Age (y)	50.5 (14.4)	52.3 (14.0)	43.8 (12.1)	0.18^‡^
Age of Psoriasis	29.9 (13.0)	20.9 (9.9)	n/a	0.04
Age of PsA	n/a	32.9 (8.9)	n/a	n/a
Psoriasis Duration (y)	20.6 (15.1)	31.4 (13.1)	n/a	0.04
PsA Duration (y)	n/a	19.4 (14.2)	n/a	n/a
PASI*	2.7 (0–23.8)	1.6 (0–6.6)	n/a	0.13
Tender Joints	n/a	1.3 (2.5)	n/a	n/a
Swollen Joints	n/a	0.3 (0.9)	n/a	n/a
NSAIDs	1 (4%)	8 (62%)	n/a	<0.001
DMARDs	1 (4%)	7 (54%)	n/a	0.001
Biologics	4 (17%)	7 (54%)	n/a	0.02

^†^ With the exception of age, p value reflects comparison of PsC vs PsA by Student’s t-test or Fisher’s Exact test.

^‡^ P value from ANOVA.

*Psoriasis Area and Severity Index; values indicate median PASI score (range); p value from Mann-Whitney U test.

### Genome-wide trends in the psoriatic disease sperm methylome

**Fig 1:**
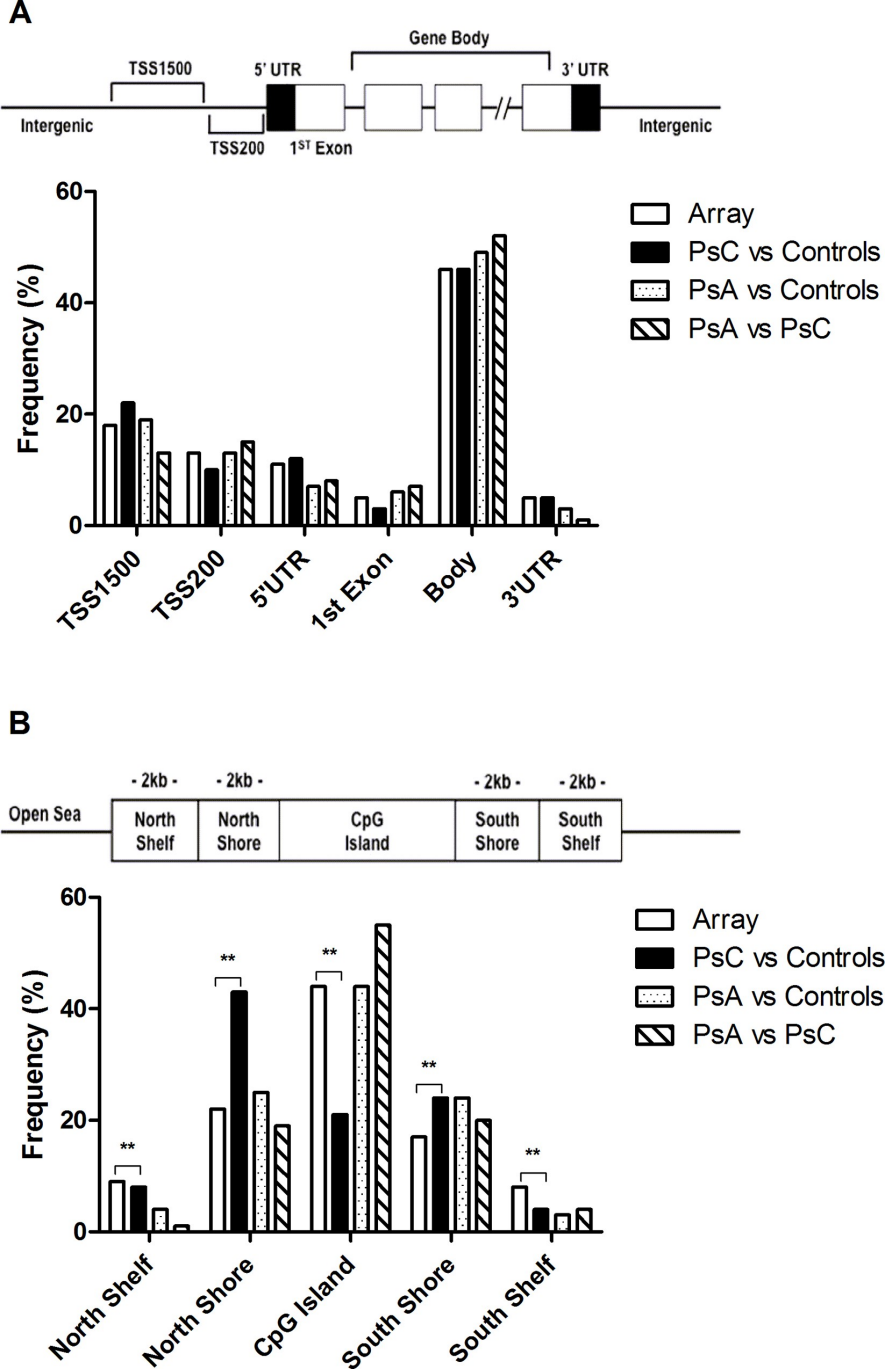
The spatial distribution of DM CpGs by gene-centric and CpG island-centric annotations. (A) No specific enrichment of DM CpG sites among gene-centric annotations was found. (B) In contrast, relative to CpG islands, DM CpG sites in PsC vs. controls were enriched in both north and south CpG island shores but depleted in north and south shelves and CpG islands (χ^2^ = 17.51, df = 4, p = 0.0015). DM CpG sites in PsA vs. controls and PsA vs. PsC were not enriched in any specific CpG island-centric annotation (χ^2^ = 5.58, df = 4, p = 0.233 and χ^2^ = 9.41, df = 4, p = 0.052, respectively).

The 331,193 CpGs assessed showed a bimodal distribution of methylation across all sperm samples analyzed, with the majority of sites showing either high methylation or low methylation levels (beta [β]>85% or <15%), consistent with previous reports [29]. Between PsC patients vs. controls there were 574 differentially methylated (DM) CpGs, 2,467 between PsA patients vs. controls, and 342 between PsA and PsC patients (mean FDR-adjusted p value [q]<0.05). The spatial distribution of these CpGs relative to genes and CpG islands is shown in Fig 1A and 1B.

**Fig 2:**
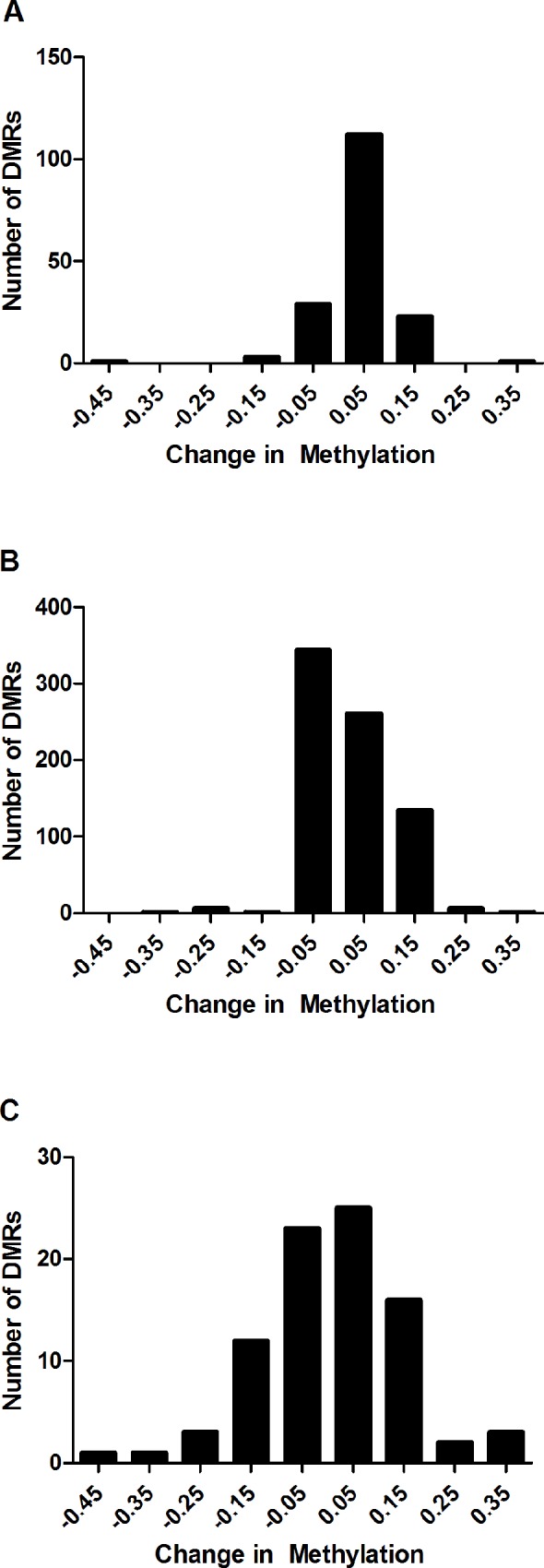
Distribution of the methylation changes observed within each set of significant DMRs. (A) PsC vs. Controls (B) PsA vs. Controls (C) PsA vs. PsC. Data is plotted in bins of 0.1 (10%) change in methylation, representing the max. beta fold change among its constituent CpG sites, with each bin indicated by its middle value (i.e. changes ranging from 0–0.1 are indicated by the bin labelled 0.05). In all 3 comparisons, the majority of DMRs represented subtle changes in methylation (from -10% hypomethylated to 10% hypermethylated). Relatively few DMRs were beyond this range in PsC probands vs. controls. Differences larger than 10% were observed in PsA probands vs. controls, which were skewed towards hypermethylation, and in PsA vs. PsC probands which were normally distributed about 0 and ranged from approximately -45% hypomethylated to 35% hypermethylated.

Significant DM CpG sites were assembled into regions of contiguous differential methylation wherever the distance to the next significant probe was less than 1kb to prioritize functionally relevant regions of methylation change. Between PsC probands vs. controls the DM CpGs grouped into 169 differentially methylated regions (DMRs) of variable size, 754 DMRs in PsA probands vs. controls, and 86 DMRs between PsA and PsC probands (mean adjusted p<0.05 of constituent CpG sites). In all 3 comparisons, the majority of DMRs showed subtle changes in methylation (from -10% hypo to 10% hypermethylated) (Fig 2A–2C).

### Differentially methylated regions in psoriatic disease are enriched in the major histocompatibility complex

Overrepresentation enrichment analysis yielded no significant enrichment of gene ontologies, pathways, networks, diseases and phenotypes among the lists of DMRs (q<0.05). However, DMRs in PsA probands vs. controls and PsC probands vs. controls were significantly enriched within the major histocompatibility complex on 6p21.3, a region which contains several loci associated with PsA and PsC, including *HLA-B*, *HLA-C*, *MICA*, *HCP5*, and *TNFA* [30]. Thirty-one DMRs between PsA probands vs. controls mapped this region (Hg19 coordinates chr6:26033821–35659141), 11 of which mapped to annotated genes (q = 4.31x10^-4^). Eighteen DMRs between PsC probands vs. controls mapped to a smaller region contained therein (hg19 coordinates chr6:28889358–33677751), 10 of which mapped to annotated genes (q = 1.36x10^-4^) (S2 Table). DMRs from both comparisons tended to occur outside the main psoriatic disease susceptibility region containing *HLA-C* and *HLA-B*. In PsA probands vs. controls, the closest DMRs to this region were found in *HCG26* and *LTB* centromeric to *HLA-B*, and near *HCG22* telomeric to *HLA-C*, while in PsC vs. controls, the closest DMRs to this region were found in *HCG26*, and just upstream of *PSORS1C1* telomeric to *HLA-C* (S2 Fig).

### Top differentially methylated regions in sperm cells contain biologically relevant genes

We prioritized DMRs for downstream analysis by limiting our analysis to those showing ≥10% change in methylation (hypo or hypermethylation). Of these, biological inferences were used to further prioritize biologically relevant sperm DMRs (Table 2). Several genes relevant to psoriatic disease pathogenesis, such as those involved in innate immunity, and skin and bone biology were identified. The top hit associated with skin disease (PsC probands vs. controls) was within the promoter of the immune-activating receptor *SIGLEC14* (sialic acid binding Ig like lectin 14), which was hypermethylated by 10% (q = 4.32x10^-6^) [31]. DMRs within the 3’UTR of junctional adhesion molecule 3 (*JAM3*), which regulates neutrophil transepithelial migration [32], and in the promoter region of procollagen-C endopeptidase enhancer (*PCOLCE*), a glycoprotein which binds and promotes the cleavage of type I procollagen to yield mature fibrillar collagen type I, a major component of the dermis, were 16% hypomethylated and 11% hypermethylated, respectively. Furthermore, a DMR within the body of retinoid X receptor beta (*RXRB*), which contains a 3'+140A polymorphism associated with chronic plaque psoriasis patients with a positive family history of disease [33], was 11% hypermethylated.

**Table 2:** Biologically relevant DMRs with ≥10% change in methylation identified in each of the 3 comparisons.

Comparison	Hg19 Coordinates	Gene	Location Relative to Gene	# CpG Sites	Mean Adj. P-val.	Max. β
**PsC vs. Controls**	chr19:52150230–52150634	*SIGLEC14*	TSS200, TSS1500	4	4.32 x10^-6^	0.10
	chr11:134019083–134019440	*JAM3*	3'UTR	6	1.54 x10^-4^	-0.16
	chr7:100199764–100200009	*PCOLCE*	TSS200, 1stExon, 5'UTR	6	2.07 x10^-4^	0.11
	chr13:111301317–111301774	*CARS2*	Body	6	7.26 x10^-4^	-0.16
	chr4:40428028–40428121	*RBM47*	Body	3	1.10 x10^-3^	0.18
	chr16:46604297–46604297	*ANKRD26P1*	TSS1500	1	1.91 x10^-3^	0.15
	chr16:69597925–69598417	*NFAT5*	TSS1500	4	2.25 x10^-3^	0.10
	chr19:5231268–5231884	*PTPRS*	Body	4	3.10 x10^-3^	0.11
	chr6:33165404–33166165	*RXRB*	Body	10	3.71 x10^-3^	0.11
	chr10:1206555–1206690	*NCRNA00200*	Body	3	4.55 x10^-3^	0.12
**PsA vs. Controls**	chr17:79792334–79793795	*DYSFIP1*	Body, TSS1500, 1stExon, 5'UTR, TSS200	14	3.66 x10^-12^	0.12
	chr10:1595543–1596108	*ADARB2*	Body	9	5.03 x10^-9^	0.21
	chr18:74728834–74729551	*MBP*	1stExon, Body, 5'UTR, TSS200, TSS1500	15	2.43 x10^-7^	0.14
	chr7:151542024–151542804	*PRKAG2*	Body	5	2.95 x10^-7^	0.15
	chr21:46349059–46349496	*ITGB2*	TSS1500	3	1.00 x10^-4^	0.37
	chr11:3186792–3188566	*OSBPL5*	TSS1500	30	1.19 x10^-4^	0.11
	chr7:47343076–47343625	*TNS3*	Body	5	1.56 x10^-4^	0.11
	chr15:25414716–25415399	*SNORD115-1*	TSS1500	7	1.63 x10^-4^	0.16
	chr6:31438939–31439497	*HCG26*	TSS200, Body	5	4.33 x10^-4^	-0.25
	chr11:2019079–2020560	*H19*, *MIR675*	TSS200, TSS1500	40	1.90x10^-3^	-0.10
**PsA vs. PsC**	chr11:34534480–34535579	*ELF5*	5'UTR, TSS1500, 1stExon, TSS200	14	1.07 x10^-8^	0.14
	chr4:7512016–7512498	*SORCS2*	Body	5	1.10 x10^-8^	0.33
	chr6:32133929–32135803	*EGFL8*	5'UTR, Body, 3'UTR	49	1.07 x10^-7^	0.15
	chr22:24372926–24374012	*LOC391322*	TSS200, Body, 3'UTR	7	5.97 x10^-4^	-0.19
	chr12:5603131–5603989	*NTF3*	TSS200, Body, 1stExon	12	1.35 x10^-3^	0.11
	chr8:1004106–1004177	*ERICH1-AS1*		2	1.86 x10^-3^	-0.24
	chr12:68647015–68647590	*IL22*	Body, 1stExon, 5'UTR, TSS200, TSS1500	8	2.02 x10^-3^	0.10
	chr10:133767507–133768327	*PPP2R2D*	Body	9	3.00 x10^-3^	-0.14
	chr19:3687751–3688477	*PIP5K1C*	Body	6	4.66 x10^-3^	0.19
	chr7:158157881–158158760	*PTPRN2*	Body	5	9.54 x10^-3^	-0.17

Top hits representing skin and/or joint disease (PsA probands vs. controls) included a 14% hypermethylated DMR spanning the promoter and body of myelin basic protein (*MBP*), an autoantigen in multiple sclerosis. The promoter of oxysterol binding protein like 5 (*OSBPL5*), a paternally imprinted gene in the placenta that encodes an intracellular lipid receptor which plays a role in maintaining cholesterol balance, was also hypermethylated by 11%. The small nucleolar RNA 115 (*SNORD115*), another paternally imprinted gene located in the Prader-Willi Syndrome (PWS) region of chromosome 15, was hypermethylated by 16%. Notably, the promoter and body of HLA complex group 26 (*HCG26*) was hypomethylated by 25%. The function of *HCG26* is unknown; however, it is located within the MHC between putative PsA risk loci *MICA* and *MICB* and lies in an intron of the adjacent locus *HCP5*, which has also been associated with PsA in GWAS.

DMRs associated with joint disease (PsA vs. PsC probands) included the promoter and body of interleukin-22 (*IL22*), a cytokine produced by IL-23-driven Th17 cells, activated γδ T cells, CD8+ T cells, and monocytes [34], which was 10% hypermethylated in PsA probands. The promoter of E74 like ETS transcription factor 5 (*ELF5*), a transcription factor expressed solely in epithelium which regulates terminal differentiation of keratinocytes, was 14% hypermethylated in PsA probands. The body of protein phosphatase 2 regulatory subunit B delta (*PPP2R2D*) was 14% hypomethylated in PsA probands. Knockout of this gene in cancer cells has been found to inhibit apoptosis and enhance proliferation and cytokine production in T cells [35]. The body of protein tyrosine phosphatase, receptor type N2 (*PTPRN2*), which contains a SNP previously associated with PsA in GWAS [36], was 17% hypomethylated in PsA probands, while the promoter and body of *HCG26* was also significantly hypomethylated by 31% in PsA vs. PsC probands.

### Pyrosequencing validates array results for several genes except *HCG26* due to a transposable element insertion

Array data were validated by pyrosequencing of the same sperm samples initially analyzed. Four DMRs representing a range of methylation differences were selected for validation (*CSMD1*, *PTDSS2*, *TPPP*, and *HCG26*). Array and pyrosequencing data were strongly correlated (Pearson’s r = 0.97) for three out of the four DMRs (*CSMD1*, *PTDSS2*, and *TPPP*, but not *HCG26* [r = 0.53] (S3 Fig). *HCG26* was found to contain a 298bp AluY transposable element insertion which is polymorphic, being present in some *HCG26* transcripts but not others [37]. Pyrosequencing of the *HCG26* DMR revealed 100% methylation of all CpG sites in all subjects, while genotyping of the AluY insertion showed a perfect correlation between zygosity and methylation measured by Infinium arrays (S3 Table), suggesting that the insertion interfered with the binding of the Infinium probe yielding inaccurate measurements on the arrays.

### Methylation of *IL22* in whole blood correlates with sperm and is independent of copy number variation

Two rounds of epigenome-wide reprogramming occur between generations, first in the blastocyst after fertilization, during which gamete-specific marks are erased to ensure totipotency, and second in early embryogenesis, during which imprinted marks are erased and re-set in the primordial germ cells depending on the sex of the embryo [38]. For a proband to inherit an epigenetic mark from a previous generation, it must become ‘imprinted-like’ and resist the first round of reprogramming [39], and for the same proband to transmit it to the next generation, it must also resist the second round. DMRs present in sperm may be explained by the fact that they were inherited from a previous generation and have resisted the first and second rounds of epigenetic reprogramming.

**Fig 3:**
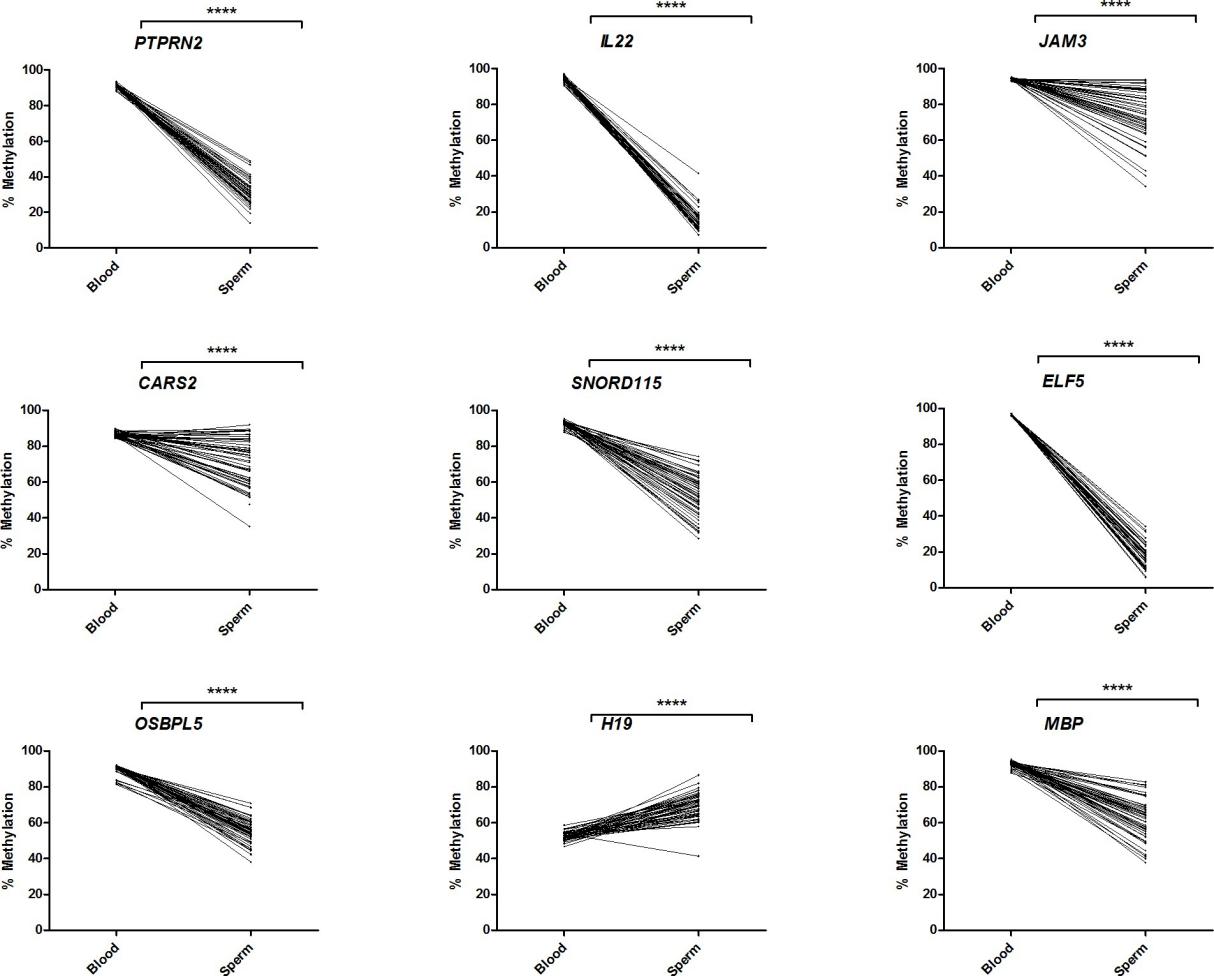
Comparison of methylation levels in blood and sperm samples. Methylation was measured in paired blood samples collected from the same individuals who provided semen samples by pyrosequencing. Differences between tissues were analyzed by paired t-test. ****, p<0.0001.

To investigate whether sperm DMRs were inherited, we measured them in whole blood, a somatic tissue, collected from the same individuals who provided semen samples. Thirteen sperm DMRs were chosen based on adjusted p-value, β difference, biological or functional relevance, and the density of significant probes within the DMR. Successful pyrosequencing assays were developed for 9 of the 13 DMRs. Due to fact that they are distinct tissues, there were large and significant differences (p<0.0001) in methylation at all DMRs in paired blood compared to sperm samples. Most DMRs, apart from *H19*, showed nearly 100% methylation (80–100%) in blood, while showing a range in methylation in sperm samples from 0–40% methylation (*IL22*, *ELF5*, and *PTPRN2*), to 40–80% methylation (*JAM3*, *CARS2*, *SNORD115*, *OBSPL5*, *H19*, and *MBP*) (Fig 3). *H19*, which is paternally imprinted and normally heavily methylated on the paternal allele, was found to have higher methylation levels in sperm cells (60–80%) compared to blood cells (~50%).

**Fig 4:**
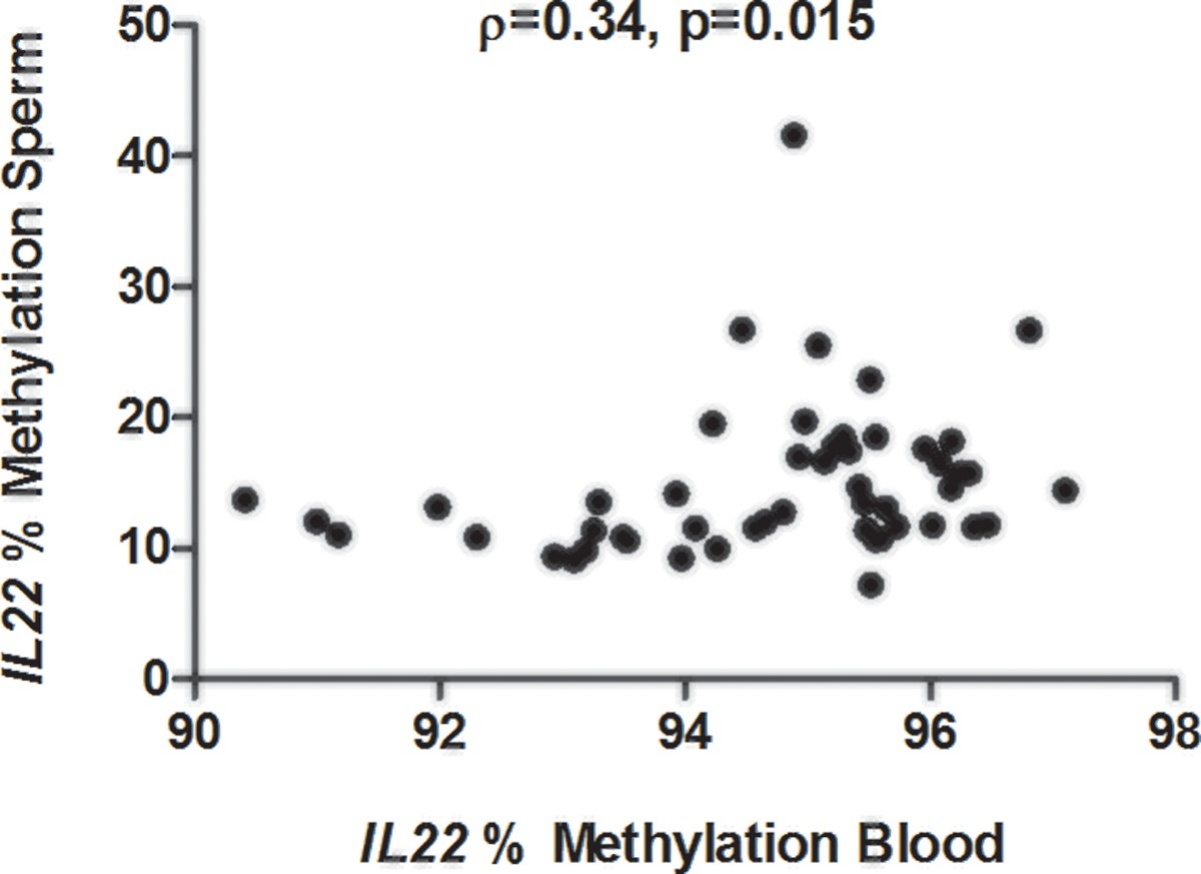
Correlation between sperm and blood methylation of *IL22*. Methylation of exon 1 of *IL22* was significantly correlated across sperm and blood samples, as measured by Infinium arrays (sperm) and pyrosequencing (blood).

Despite these tissue-specific differences, there was a significant positive correlation between sperm and blood methylation for *IL22* (ρ = 0.34 [95% CI 0.06–0.57], p = 0.015, Fig 4), but not the other loci (S4 Table). Exon 1 of *IL22* contains a CNV previously associated with psoriasis vulgaris [40]. This CNV encompasses the binding site of an Infinium probe within the *IL22* DMR. Due to the potential of CNVs to bias methylation measurements, the *IL22* exon 1 CNV was genotyped in all sperm and blood samples. No variation in copy number was found (all samples contained two copies, S5 Table), indicating that *IL22* methylation cannot be attributed to the underlying CNV and reflect true methylation levels at this locus.

## Discussion

Despite several large-scale efforts to identify genetic underpinnings of psoriatic disease, much of its heritability remains unexplained. Here, we explored whether epigenetic factors contribute to its heritability by taking the novel approach of genome-wide methylation profiling of sperm cells. An advantage of beginning our investigation with sperm cells is that they were easily attainable and could be purified into a homogeneous cell preparation, allowing us to avoid the confounding effects of cellular heterogeneity.

As sperm cells of psoriasis and PsA patients have never been studied, we were interested in large genomic and small-scale differences between patients and controls. One study of human sperm cells found that the largest degree of epigenetic variation occurs at functionally important promoter-associated CpG islands [31]. When associated with transcriptional start sites, CpG island methylation is associated with long-term gene silencing such as imprinting, or with genes expressed predominantly in germ cells and some tissue-specific genes [33]. We similarly found that the DM CpG sites in PsC vs. controls were enriched in such regions, as well as in north and south CpG island shores—areas whose functional relevance is still unclear [33].

DM CpG sites were assembled into larger DMRs to aid in identifying functionally relevant regions of methylation change [27]. As this effectively averages methylation across large regions, DMRs were overall quite subtle between groups, with most changes <10% in each comparison. Other studies comparing methylation at CpG site level have also noted that although there is considerable epigenetic variation in human sperm cells [41], these variations are quite subtle, or are present in very low frequencies of cells (<1%) [41, 42], consistent with our observations. Although few DMRs exceeded a 10% change in methylation, it is noteworthy that many of those which did play roles in the pathogenesis of skin disease (*SIGLEC14*, *JAM3*, *PCOLCE*, *RXRB*, *ELF5*, *IL22*), joint disease (*MBP*, *HCG26*, *IL22*, *PPP2R2D*, *PTPRN2*) or are known to be imprinted (*OSBPL5*, *SNORD115*) (Table 2). DMRs were also found to be enriched within the MHC, but were generally not in close proximity to the Class I psoriatic disease susceptibility loci *HLA-C* and *HLA-B*. Whether these DMRs represent distal regulatory elements for known psoriatic disease susceptibility loci remains to be determined.

We investigated candidate DMRs in blood to provide further evidence that they were inherited, but also to determine if observations in sperm can be translated to blood, a more pathogenically relevant tissue. The finding of *IL22* as significantly hypermethylated in sperm of PsA compared to PsC probands and its significant correlation with methylation levels in the blood is particularly interesting given its role in inflammatory skin and joint disease. IL-22 is an IL-10 family cytokine that is secreted by a diverse repertoire of cells including Th1, Th17, Th22, and NK cells in response to IL-23. It is recognized by a heterodimeric receptor consisting of IL-10R2 and IL-22R1, with the latter being expressed specifically in lung, gastrointestinal, and skin epithelia but not on immune cells, making IL-22 a terminal effector cytokine [43]. IL-22 is suspected to play an important role in psoriatic skin disease by inducing hyperplasia and migration of epidermal keratinocytes, and expression of antimicrobial peptides, suggesting a role in wound healing and antimicrobial responses [43, 44]. It is highly overexpressed in psoriatic skin lesions and blood of psoriasis patients, and expression correlates with disease severity [45]. In mice, IL-22 is highly expressed in the entheses, and has been found to promote entheseal and periosteal bone formation [46]. Furthermore, IL-22 is expressed at higher levels in PsA compared to OA synovial fluid, and activated T cells from the synovium of PsA patients produce more IL-22 than OA patients, suggesting a role for IL-22 in PsA [47]. In this context, our finding of *IL22* hypermethylation seems paradoxical given that it is usually associated with transcriptional repression. However, the DMR identified encompassed the gene body, which can be associated with moderate expression [48]. Unfortunately, we could not assess expression of *IL22* in whole blood of the probands tested due to its low expression level in unstimulated *ex vivo* cells. Future studies will aim to validate *IL22* hypermethylation and correlate with expression in stimulated cells to demonstrate the functional consequences of the identified epigenetic variants on transcription.

Although a DMR within the first exon of *IL22* showed a significant correlation between blood and sperm cells, which is independent of an overlapping CNV in *IL22*, at present we cannot rule out the possibility that other genetic variants in *IL-22* affect methylation of this DMR. Sequencing studies are necessary to demonstrate that methylation is independent of *cis* or *trans*-acting genetic mutations. If methylation is indeed independent of genetic variation in *IL-22*, it may be considered a putative primary epimutation warranting further study. For example, it will be necessary to know whether these germ line variants are present in normal somatic tissues derived from the three germ layers (ectoderm, mesoderm, and endoderm), as this would suggest that it is a ‘constitutional’ epimutation derived from the germ line and present in all tissues [18], lending further support for inheritance from the previous generation. Similarly, it would also be helpful to demonstrate the presence of these variants in the germ line of the parents, or the somatic tissues of the offspring of the patients studied here.

## Conclusions

This study provides preliminary evidence of epigenetic variations in human sperm cells that are associated with PsC and PsA. These variations are generally subtle but occur near or within several genes that have potential pathogenic relevance. We identified a hypermethylated DMR within the first exon of *IL22* which was correlated between sperm and whole blood as a putative epimutation associated with joint disease, but further studies are needed to rule out an underlying genetic cause and determine if this is widely distributed in somatic cells, which would suggest a heritable, constitutional epimutation. We also identified several other DMRs between psoriasis patients, PsA patients, and controls which may be the result of exposure of germ cells to endogenous or exogenous environmental factors.

## Supporting information

S1 Table PCR primers for amplifying the 298bp AluY insertion in *HCG26*. (PDF)

S2 Table Enriched DMRs between PsA probands vs. controls and PsC probands vs. controls in the MHC that map to annotated genes. (PDF)

S3 Table Genotyping results of the AluY insertion in *HCG26* and corresponding Infinium array results. 0 copies of the insertion results in >90% methylation at the *HCG26* locus, 1 copy results in a readout of ~55% methylation, while 2 copies results in a readout of ~20% methylation.
(PDF)

S4 Table Correlation between sperm and whole blood methylation across candidate DMRs. (PDF)

S5 Table IL22 *CNV* genotyping results in blood and sperm samples. (PDF)

S1 Fig Frequency of p values for the association of clinical, demographic, and technical characteristics with sperm CpG methylation. (PDF)

S2 Fig Genetic map of DMRs in PsA and PsC probands vs. controls enriched in the MHC on 6p21.3. Top-bottom, high-level to detailed view of chromosome 6 showing enrichment within the MHC. Dashes indicate location of DMRs between PsA probands vs. controls (red) and PsC probands vs. controls (blue). Bar graphs depict beta fold change and–log10 of the q values for each DMR. Green box indicates psoriatic disease susceptibility region including *HLA-C* and *HLA-B*.
(PDF)

S3 Fig Technical validation of arrays in sperm samples. Correlation between Infinium % methylation and gold-standard bisulfite pyrosequencing (x = y included for comparison).
(PDF)
