# Supplementary material for: Epigenome-wide analysis of sperm cells identifies IL22 as a possible germ line risk locus for psoriatic arthritis
Source: PLoS One. 2019 Feb 19;14(2):e0212043. doi: 10.1371/journal.pone.0212043 (PMC6380582; doi:10.1371/journal.pone.0212043)
Supplement: S1 Table — (PDF) [file pone.0212043.s001.pdf]

S1 Table. PCR primers for amplifying the 298bp AluY insertion in *HCG26*.

F Primer (5’->3’)	GCGATGCATCCTCTCTGCT
R Primer (5’->3’)	GGACAGAGCCCAGGTGAAAG
