# Supplementary material for: Epigenome-wide analysis of sperm cells identifies IL22 as a possible germ line risk locus for psoriatic arthritis
Source: PLoS One. 2019 Feb 19;14(2):e0212043. doi: 10.1371/journal.pone.0212043 (PMC6380582; doi:10.1371/journal.pone.0212043)
Supplement: S2 Table — (PDF) [file pone.0212043.s002.pdf]

S2 Table. Enriched DMRs between PsA probands vs. controls and PsC probands vs. controls in the MHC (6p21.3) that map to annotated genes.

**PsA probands vs. Controls (enrichment q=4.31x10^{-4})**	
**Gene Symbol**	**Gene Name**
EHMT2	euchromatic histone lysine methyltransferase 2
NRM	nurim (nuclear envelope membrane protein)
TUBB	tubulin beta class I
LTB	lymphotoxin beta
NOTCH4	notch 4
PSMB8	proteasome subunit beta 8
TNXB	tenascin XB
TRIM26	tripartite motif containing 26
OR12D3	olfactory receptor family 12 subfamily D member 3
TRIM15	tripartite motif containing 15
PHF1	PHD finger protein 1
**PsC probands vs. Controls (enrichment q=1.36x10^{-4})**	
HCP5	HLA complex P5 (non-protein coding)
EHMT2	euchromatic histone lysine methyltransferase 2
GABBR1	gamma-aminobutyric acid type B receptor subunit 1
PSMB8	proteasome subunit beta 8
TRIM27	tripartite motif containing 27
HSD17B8	hydroxysteroid 17-beta dehydrogenase 8
C6orf25	megakaryocyte and platelet inhibitory receptor G6b
ZBTB22	zinc finger and BTB domain containing 22
ZBTB9	zinc finger and BTB domain containing 9
CUTA	cutA divalent cation tolerance homolog
