# Supplementary material for: Epigenome-wide analysis of sperm cells identifies IL22 as a possible germ line risk locus for psoriatic arthritis
Source: PLoS One. 2019 Feb 19;14(2):e0212043. doi: 10.1371/journal.pone.0212043 (PMC6380582; doi:10.1371/journal.pone.0212043)
Supplement: S3 Table — 0 copies of the insertion results in >90% methylation at the HCG26 locus, 1 copy results in a readout of ~55% methylation, while 2 copies results in a readout of ~20% methylation. (PDF) [file pone.0212043.s003.pdf]

S2 Table. Genotyping results of the AluY insertion in *HCG26* and corresponding Infinium array results. 0 copies of the insertion results in >90% methylation at the *HCG26* locus, 1 copy results in a readout of ~55% methylation, while 2 copies results in a readout of ~20% methylation.

**Group**	**Sample**	**Infinium *HCG26* Beta Value (Sperm)**	**PCR Genotyping HCG26-AluYIns**
Control	1	0.55	1
Control	2	0.56	1
Control	3	0.53	1
Control	4	0.93	0
Control	5	0.94	0
Control	6	0.93	0
Control	7	0.56	1
Control	8	0.93	0
Control	9	0.53	1
Control	10	0.55	1
Control	11	0.93	0
Control	12	0.52	1
Control	13	0.52	1
PsA	1	0.52	1
PsA	2	0.57	1
PsA	3	0.55	1
PsA	4	0.27	2
PsA	5	0.59	1
PsA	6	0.51	1
PsA	7	0.18	2
PsA	8	0.93	0
PsA	9	0.56	1

PsA	10	0.57	1
PsA	11	0.56	1
PsC	1	0.93	0
PsC	2	0.57	1
PsC	3	0.51	1
PsC	4	0.93	0
PsC	5	0.93	0
PsC	6	0.92	0
PsC	7	0.54	1
PsC	8	0.6	1
PsC	9	0.23	2
PsC	10	0.94	0
PsC	11	0.92	0
PsC	12	0.94	0
PsC	13	0.49	1
PsC	14	0.56	1
PsC	15	0.56	1
PsC	16	0.93	0
PsC	17	0.93	0
PsC	18	0.91	0
PsC	19	0.92	0
PsC	20	0.94	0
