# Supplementary material for: Epigenome-wide analysis of sperm cells identifies IL22 as a possible germ line risk locus for psoriatic arthritis
Source: PLoS One. 2019 Feb 19;14(2):e0212043. doi: 10.1371/journal.pone.0212043 (PMC6380582; doi:10.1371/journal.pone.0212043)
Supplement: S4 Table — (PDF) [file pone.0212043.s004.pdf]

S3 Table. Correlation between sperm and whole blood methylation across candidate DMRs.

Gene	Spearman correlation coefficient (95% CI) (Sperm vs. Whole Blood)	P-Value
CARS2	-0.04 (-0.32-0.26)	0.81
ELF5	0.13 (-0.17-0.40)	0.38
H19	0.03 (-0.26-0.31)	0.84
IL22	**0.34 (0.06-0.57)**	**0.015**
JAM3	-0.28 (-0.52-0.0007)	0.05
MBP	0.01 (-0.28-0.30)	0.94
OSBPL5	0.20 (-0.09-0.46)	0.17
PTPRN2	0.10 (-0.19-0.38)	0.48
SNORD115	-0.04 (-0.32-0.25)	0.80
