# Supplementary material for: Epigenome-wide analysis of sperm cells identifies IL22 as a possible germ line risk locus for psoriatic arthritis
Source: PLoS One. 2019 Feb 19;14(2):e0212043. doi: 10.1371/journal.pone.0212043 (PMC6380582; doi:10.1371/journal.pone.0212043)
Supplement: S5 Table — (PDF) [file pone.0212043.s005.pdf]

**S4 Table.** ***IL22*** **CNV genotyping results in blood and sperm samples.**

| Sample | Group | Sample Type | IL22 Copy # Calculated | IL22 Copy Number Predicted | Confidence |
|---|---|---|---|---|---|
| 1 | CTL | Blood | 1.66 | 2 | 0.98 |
| 2 | CTL | Blood | 1.81 | 2 | 0.95 |
| 3 | CTL | Blood | 1.95 | 2 | 0.99 |
| 4 | CTL | Blood | 1.88 | 2 | 0.98 |
| 5 | CTL | Blood | 1.7 | 2 | 0.98 |
| 6 | CTL | Blood | 1.99 | 2 | 0.95 |
| 7 | CTL | Blood | 2.16 | 2 | 0.99 |
| 8 | CTL | Blood | 2.02 | 2 | 0.99 |
| 9 | CTL | Blood | 2.09 | 2 | 0.97 |
| 10 | CTL | Blood | 2.2 | 2 | 0.97 |
| 11 | CTL | Blood | 2.12 | 2 | 0.99 |
| 12 | CTL | Blood | 2.2 | 2 | 0.98 |
| 1 | PS | Blood | 1.89 | 2 | 0.98 |
| 2 | PS | Blood | 1.78 | 2 | 0.98 |
| 3 | PS | Blood | 2.07 | 2 | 0.95 |
| 4 | PS | Blood | 1.98 | 2 | 0.96 |
| 5 | PS | Blood | 1.75 | 2 | 0.995 |
| 6 | PS | Blood | 1.78 | 2 | 0.96 |
| 7 | PS | Blood | 2.01 | 2 | 0.98 |
| 8 | PS | Blood | 1.77 | 2 | 0.995 |
| 9 | PS | Blood | 1.88 | 2 | 0.96 |
| 10 | PS | Blood | 1.98 | 2 | 0.97 |
| 11 | PS | Blood | 2.1 | 2 | 0.95 |
| 12 | PS | Blood | 1.85 | 2 | 0.99 |
| 13 | PS | Blood | 1.9 | 2 | 0.99 |

| | | | | | |
|---|---|---|---|---|---|
| 14 | PS | Blood | 1.83 | 2 | 0.97 |
| 15 | PS | Blood | 1.8 | 2 | 0.995 |
| 16 | PS | Blood | 1.99 | 2 | 0.98 |
| 17 | PS | Blood | 1.83 | 2 | 0.98 |
| 18 | PS | Blood | 1.81 | 2 | 0.995 |
| 19 | PS | Blood | 2.11 | 2 | 0.94 |
| 20 | PS | Blood | 1.88 | 2 | 0.97 |
| 21 | PS | Blood | 2.02 | 2 | 0.96 |
| 22 | PS | Blood | 1.98 | 2 | 0.98 |
| 1 | PSA | Blood | 1.71 | 2 | 0.995 |
| 2 | PSA | Blood | 1.97 | 2 | 0.97 |
| 3 | PSA | Blood | 2.01 | 2 | 0.95 |
| 4 | PSA | Blood | 1.95 | 2 | 0.97 |
| 5 | PSA | Blood | 2.01 | 2 | 0.96 |
| 6 | PSA | Blood | 2.02 | 2 | 0.95 |
| 7 | PSA | Blood | 1.94 | 2 | 0.98 |
| 8 | PSA | Blood | 2.1 | 2 | 0.97 |
| 9 | PSA | Blood | 2.03 | 2 | 0.96 |
| 10 | PSA | Blood | 1.83 | 2 | 0.995 |
| 1 | CTL | Sperm | 1.92 | 2 | 0.97 |
| 2 | CTL | Sperm | 1.83 | 2 | 0.995 |
| 3 | CTL | Sperm | 2.19 | 2 | 0.99 |
| 4 | CTL | Sperm | 1.9 | 2 | 0.96 |
| 5 | CTL | Sperm | 1.9 | 2 | 0.93 |
| 6 | CTL | Sperm | 2.16 | 2 | 0.995 |
| 7 | CTL | Sperm | 1.87 | 2 | 0.96 |
| 8 | CTL | Sperm | 1.78 | 2 | 0.995 |

| | | | | | |
|---|---|---|---|---|---|
| 9 | CTL | Sperm | 1.82 | 2 | 0.98 |
| 10 | CTL | Sperm | 1.8 | 2 | 0.97 |
| 11 | CTL | Sperm | 1.95 | 2 | 0.98 |
| 12 | CTL | Sperm | 1.82 | 2 | 0.98 |
| 13 | CTL | Sperm | 1.87 | 2 | 0.995 |
| 14 | CTL | Sperm | 2.18 | 2 | 0.99 |
| 15 | CTL | Sperm | 1.85 | 2 | 0.995 |
| 16 | CTL | Sperm | 2.31 | 2 | 0.9 |
| 1 | PS | Sperm | 2.23 | 2 | 0.95 |
| 2 | PS | Sperm | 1.94 | 2 | 0.98 |
| 3 | PS | Sperm | 1.68 | 2 | 0.97 |
| 4 | PS | Sperm | 1.9 | 2 | 0.96 |
| 5 | PS | Sperm | 1.84 | 2 | 0.995 |
| 6 | PS | Sperm | 1.88 | 2 | 0.98 |
| 7 | PS | Sperm | 1.94 | 2 | 0.995 |
| 8 | PS | Sperm | 1.93 | 2 | 0.995 |
| 9 | PS | Sperm | 1.85 | 2 | 0.99 |
| 10 | PS | Sperm | 2.22 | 2 | 0.99 |
| 11 | PS | Sperm | 2.06 | 2 | 0.95 |
| 12 | PS | Sperm | 2.26 | 2 | 0.99 |
| 13 | PS | Sperm | 2.03 | 2 | 0.97 |
| 14 | PS | Sperm | 1.83 | 2 | 0.995 |
| 15 | PS | Sperm | 1.79 | 2 | 0.995 |
| 16 | PS | Sperm | 1.94 | 2 | 0.96 |
| 17 | PS | Sperm | 1.69 | 2 | 0.99 |
| 18 | PS | Sperm | 1.64 | 2 | 0.995 |
| 19 | PS | Sperm | 2.04 | 2 | 0.97 |

| | | | | | |
|---|---|---|---|---|---|
| 20 | PS | Sperm | 2.29 | 2 | 0.98 |
| 21 | PS | Sperm | 2.23 | 2 | 0.95 |
| 22 | PS | Sperm | 2.05 | 2 | 0.97 |
| 23 | PS | Sperm | 2.26 | 2 | 0.98 |
| 1 | PSA | Sperm | 1.89 | 2 | 0.99 |
| 2 | PSA | Sperm | 1.74 | 2 | 0.95 |
| 3 | PSA | Sperm | 1.93 | 2 | 0.97 |
| 4 | PSA | Sperm | 2.26 | 2 | 0.99 |
| 5 | PSA | Sperm | 2.11 | 2 | 0.96 |
| 6 | PSA | Sperm | 2.23 | 2 | 0.96 |
| 7 | PSA | Sperm | 2.08 | 2 | 0.995 |
| 8 | PSA | Sperm | 2.15 | 2 | 0.95 |
| 9 | PSA | Sperm | 1.88 | 2 | 0.99 |
| 10 | PSA | Sperm | 2.32 | 2 | 0.97 |
