# Supplementary material for: Epigenome-wide analysis of sperm cells identifies IL22 as a possible germ line risk locus for psoriatic arthritis
Source: PLoS One. 2019 Feb 19;14(2):e0212043. doi: 10.1371/journal.pone.0212043 (PMC6380582; doi:10.1371/journal.pone.0212043)

S1 Fig. **Frequency of p values for the association of clinical, demographic, and technical characteristics with sperm CpG methylation.**

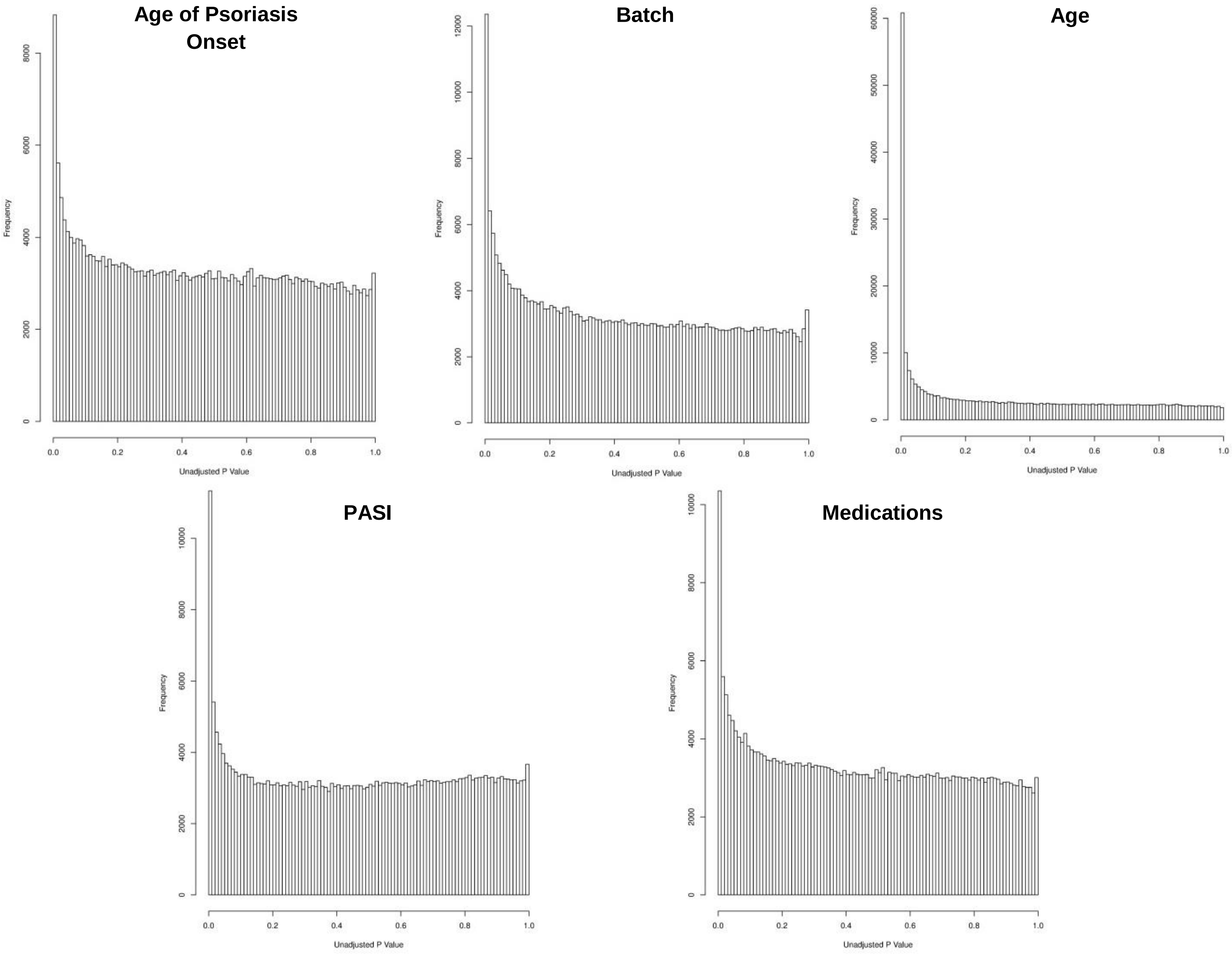

Supplement: S1 Fig — (PDF) [file pone.0212043.s006.pdf]
