# Supplementary figures and images for: Epigenome-wide analysis of sperm cells identifies IL22 as a possible germ line risk locus for psoriatic arthritis
Source: PLoS One. 2019 Feb 19;14(2):e0212043. doi: 10.1371/journal.pone.0212043 (PMC6380582; doi:10.1371/journal.pone.0212043)

Chromosome 6

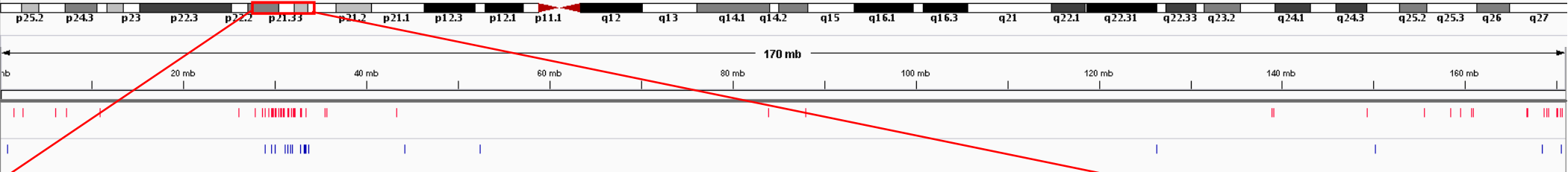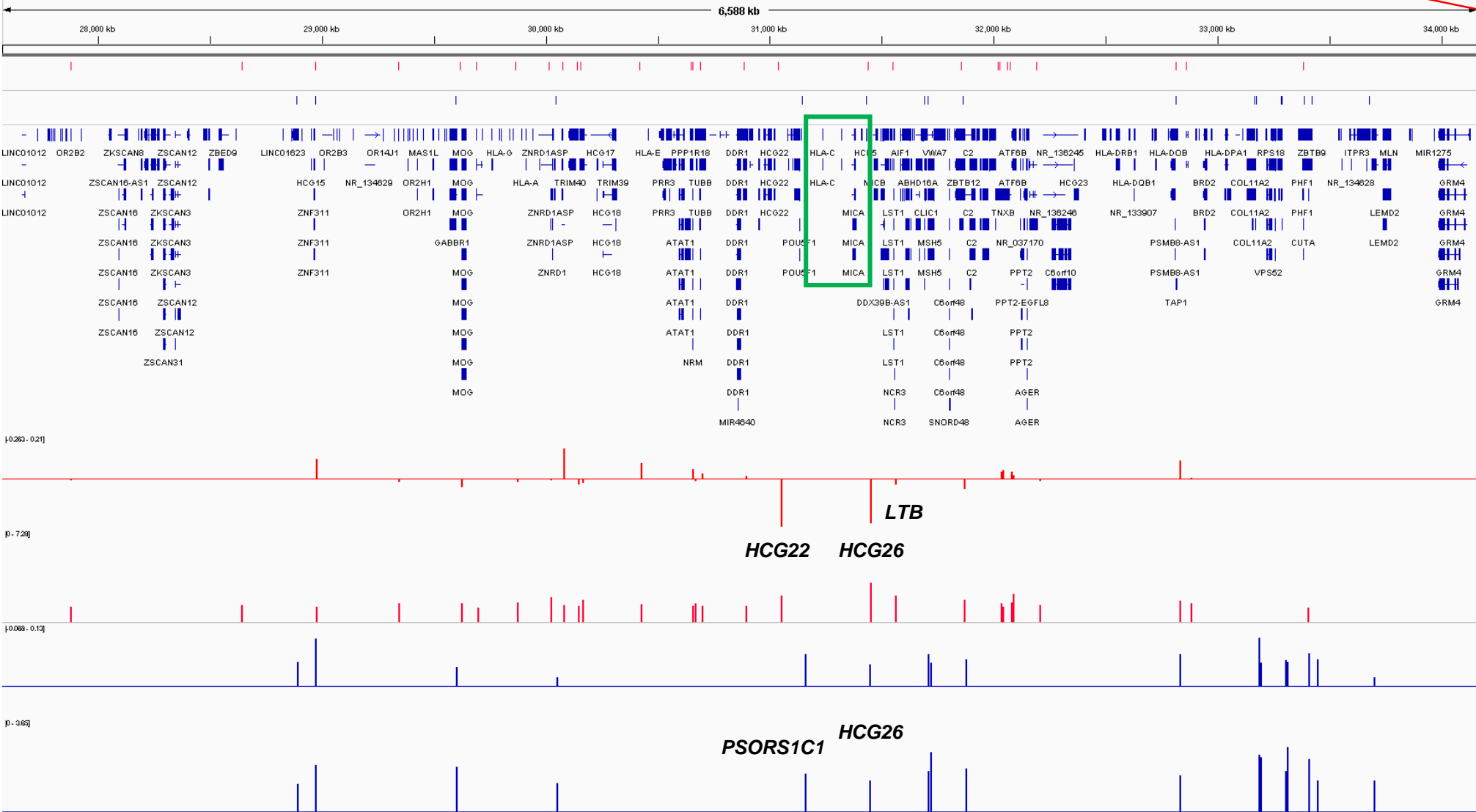

Supplement: S2 Fig — Top-bottom, high-level to detailed view of chromosome 6 showing enrichment within the MHC. Dashes indicate location of DMRs between PsA probands vs. controls (red) and PsC probands vs. controls (blue). Bar graphs depict beta fold change and–log10 of the q values for each DMR. Green box indicates psoriatic disease susceptibility region including HLA-C and HLA-B. (PDF) [file pone.0212043.s007.pdf]
