# Supplementary material for: Epigenome-wide analysis of sperm cells identifies IL22 as a possible germ line risk locus for psoriatic arthritis
Source: PLoS One. 2019 Feb 19;14(2):e0212043. doi: 10.1371/journal.pone.0212043 (PMC6380582; doi:10.1371/journal.pone.0212043)

S3 Fig. Technical validation of arrays in sperm samples. Correlation between Infinium % methylation and gold-standard bisulfite pyrosequencing (x=y included for comparison).

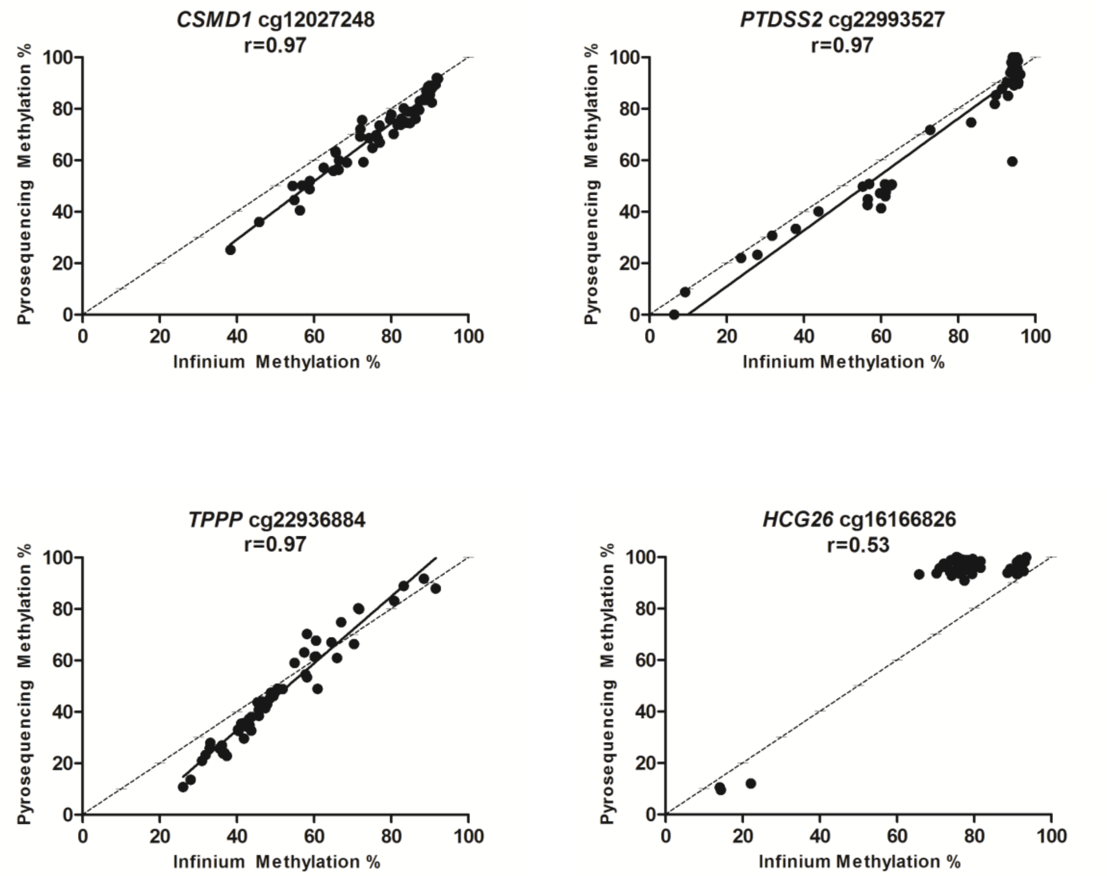

Supplement: S3 Fig — Correlation between Infinium % methylation and gold-standard bisulfite pyrosequencing (x = y included for comparison). (PDF) [file pone.0212043.s008.pdf]
